# The H*d*, H*j,* and H*z66* flagella variants of *Salmonella* *enterica* serovar Typhi modify host responses and cellular interactions

**DOI:** 10.1038/srep07947

**Published:** 2015-01-22

**Authors:** Fernanda Schreiber, Sally Kay, Gad Frankel, Simon Clare, David Goulding, Esther van de Vosse, Jaap T. van Dissel, Richard Strugnell, Guy Thwaites, Robert A. Kingsley, Gordon Dougan, Stephen Baker

**Affiliations:** 1 The Wellcome Trust Sanger Institute, Hinxton, Cambridge, United Kingdom; 2 Centre for Molecular Microbiology and Infection, Imperial College, London, United Kingdom; 3 Department of Infectious Diseases, Leiden University Medical Center, Leiden, the Netherlands; 4 Department of Microbiology and Immunology, University of Melbourne, Parkville, Australia; 5 The Hospital for Tropical Diseases, Wellcome Trust Major Overseas Programme, Oxford University Clinical Research Unit, Ho Chi Minh City, Vietnam; 6 Centre for Tropical Medicine, Nuffield Department of Clinical Medicine, Oxford University, United Kingdom; 7 Institute for Food Research, Norwich, United Kingdom; 8 The London School of Hygiene and Tropical Medicine, London, United Kingdom

## Abstract

*Salmonella* Typhi, the causative agent of typhoid fever, is a monophyletic, human-restricted bacterium that exhibits limited phenotypic variation. *S*. Typhi from Indonesia are a notable exception, with circulating strains expressing diverse flagella antigens including H*j,* H*d* and H*z66*. Hypothesizing that *S*. Typhi flagella plays a key role during infection, we constructed an *S*. Typhi *fliC* mutant and otherwise isogenic *S*. Typhi strains expressing the H*j,* H*d*, H*z66* flagella antigens. Phenotyping revealed differences in flagellum structure, strain motility and immunogenicity, but not in the ability of flagellated isolates to induce TLR5 activity. Invasion assays using epithelial and macrophage cell lines revealed differences in the ability of these *S*. Typhi derivatives to invade cells or induce cellular restructuring in the form of ruffles. Notably, the H*j* variant induced substantial ruffles that were not fully dependent on the GTPases that contribute to this process. These data highlight important differences in the phenotypic properties of *S*. Typhi flagella variation and how they impact on the pathogenesis of *S*. Typhi.

Typhoid fever, the disease caused by the bacterium *Salmonella* *enterica* serovar Typhi (*S*. Typhi), remains common in locations with poor sanitation^1^. The clinical syndrome of typhoid, with the characteristically high fever, is induced by the infecting bacteria invading the gastrointestinal surface and spreading systemically in the bloodstream^2^. The disease is seldom fatal if treated with appropriate antimicrobials but can become life-threatening, with some patients developing complications, such as intestinal perforation and neurological symptoms^3^.

*S*. Typhi is routinely identified and classified by the Kauffman-White scheme using specific typing sera^4^,^5^. The major typing antigens for *S*. Typhi are the O9 and O12 (epitopes present on the O antigen side-chain of LPS), the Vi or virulence-associated polysaccharide capsule, and the flagella (H) antigen, predominantly of type H*d*. *S*. Typhi is a monophyletic, human-restricted pathogen, and all extant organisms originate from a single common ancestor that crossed into the human population thousands of years ago^6^. Consequently, the genomes of individual *S.* Typhi are highly conserved, exhibiting limited evidence of recombination, isolate specific horizontal gene transfer, or geographically restricted pathovars^7^.

Flagellin is the monomer of the flagella filament, the dominant protein of a complex super-molecular structure, the flagellum, which is essential for bacterial motility and chemotaxis^8^,^9^. Flagellin is a key trigger of the immune response since this polypeptide engages both the innate, through Toll-like receptor 5 (TLR5), and the adaptive arm of the mammalian immune system^10^,^11^. The majority of *Salmonella* serovars are biphasic, and in a process called phase variation exhibit the ability to switch expression between two alternative flagellin genes, *fliC* and *fljB,* which encode the phase 1 and 2 flagella^8^. *Salmonella* flagella phase variation is controlled by the invertible promoter (*hin*), which influences the pattern of *fliC* and *fljB* transcription. FljA is a repressor of *fliC* transcription, ensuring that only a single flagellin gene is expressed at one time. *S.* Typhi is atypical with respect to most *Salmonella* in that it generally possesses the *fliC* H*d* encoding flagellin gene only. However, *S*. Typhi variants originating in Indonesia harbor pBSSB1, a linear plasmid encoding a *fljB* analogue that directs the expression of the H*z66* flagellin antigen^12^,^13^. Plasmid pBSSB1 additionally encodes a repressor of the chromosomal *fliC* gene; ensuring only one flagella antigen is expressed at a time. A third antigenic variant of flagellin, known as H*j*, is also found in some *S*. Typhi that also originate from Indonesia. H*j* is encoded by an allele of *fliC* gene harboring a 261 bp in-frame deletion in the central region of the H*d fliC* coding sequence^14^.

The impact of flagella antigenic variation on pathogenesis and immunity within *S*. Typhi is not well described. Furthermore, we hypothesized that flagella variation plays a unique role in regulating the immune response to *S*. Typhi infection in Indonesia, a location where a range of atypical *S*. Typhi flagella variants circulate. Here, we have engineered an aflagellated *S*. Typhi Δ*fliC* mutant and a set of three otherwise isogenic derivatives of *S.* Typhi that differ only in the flagella antigen variant expressed on the surface (H*d*, H*j* and H*z66*). These isogenic *S*. Typhi derivatives were subjected to a range of phenotypic assays including their ability to interact with epithelial cells and macrophages. We show that the flagella type can influence the immune response during typhoid and impact on the ability of *S*. Typhi to invade host cells.

## Results

### Construction and primary characterization of isogenic S. Typhi flagella variants

**Figure 1:**
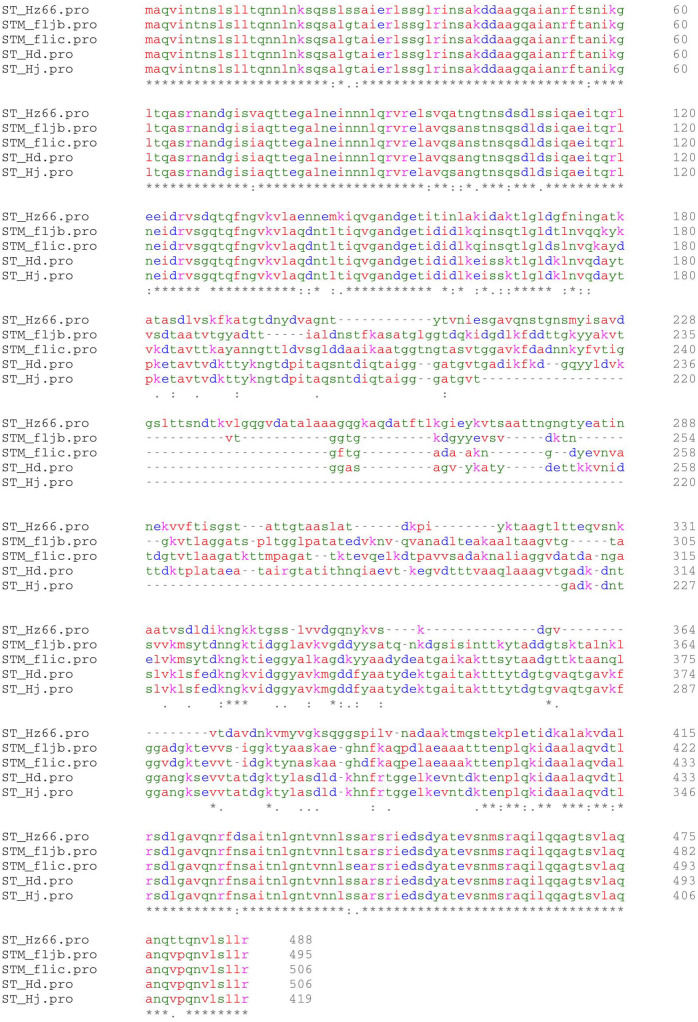
Amino acid sequence alignment of *S*. Typhi and *S*. Typhimurium flagellin proteins. Amino acid alignment of the flagellin genes (from top to bottom) FliC H*z66* (*S*. Typhi), FljB (*S*. Typhimurium), FliC (*S*. Typhimurium), FliC H*d* (*S*. Typhi) and FliC H*j* (*S*. Typhi).

**Figure 2:**
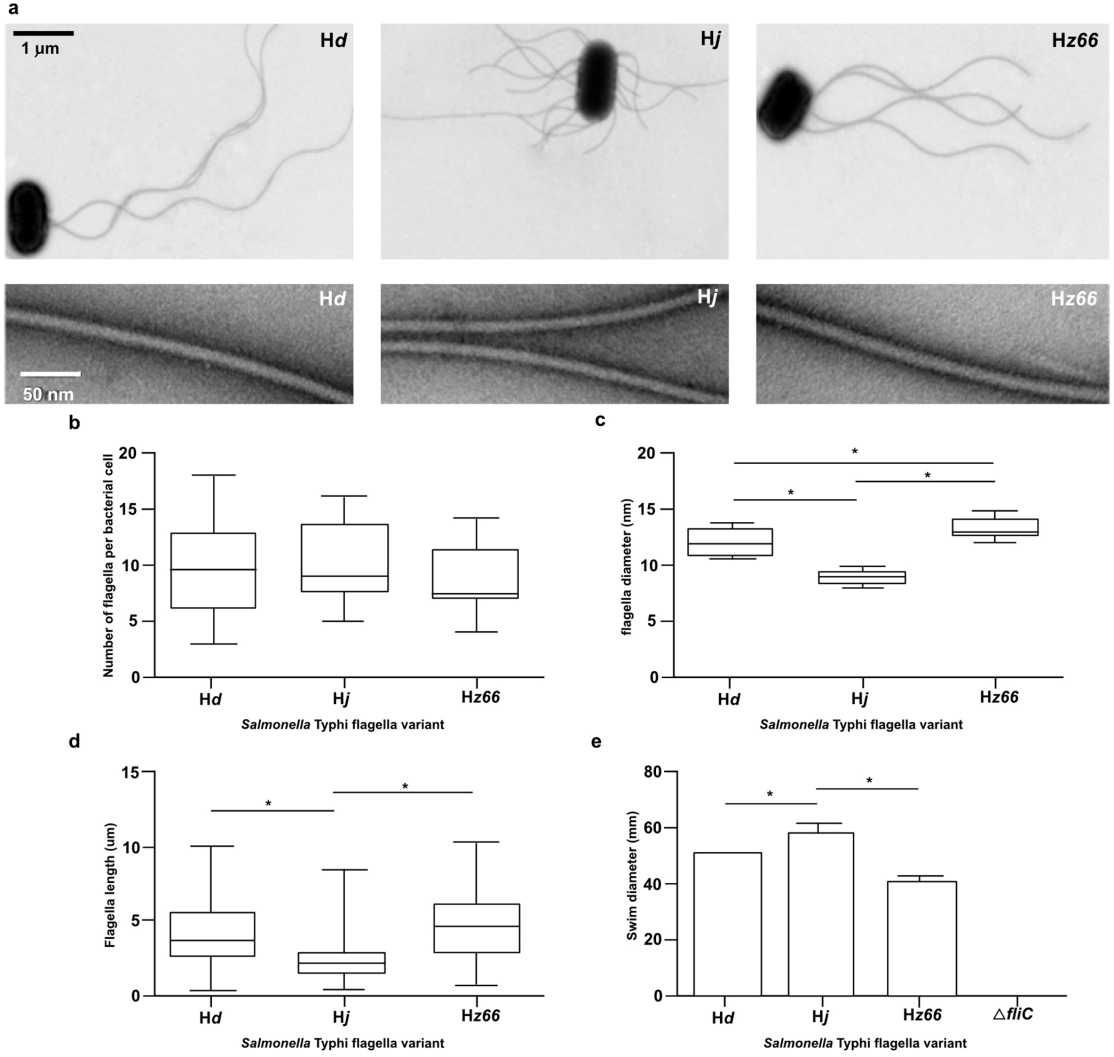
The phenotypic characteristics of *S*. Typhi flagella derivatives. (a) The three *S*. Typhi flagella derivatives (H*d*, H*j*, and H*z66*) compared by TEM to assess flagella morphology, orientation (upper panel), and width (lower panel). (b) Boxplots showing the median number of flagella per bacteria cell. (c) Boxplots showing median flagella diameter (nm). (d) Boxplots showing median flagella length (μm). (e) Histogram showing the median swim distance in soft agar of the three flagella variants over a 16-hour period. Boxes and whiskers show the interquartile ranges and range respectively. An asterisk highlights statistically significant variations by pairwise comparison (*p*-value<0.05).

To compare the properties of the H*d*, H*j* and H*z66* flagella, we used targeted mutagenesis to construct three otherwise isogenic *S.* Typhi derivatives that differed only in terms of their flagellin gene content (alignments of the flagellin proteins from *S*. Typhi and *S*. Typhimurium are shown in Figure 1). For consistency, all gene replacements targeted the *fliC* locus, i.e. the H*d* encoding *fliC* gene was completely replaced with only the H*j* *fliC* or the H*z66* *fljB* allele using the native *fliC* promoter to direct expression. An additional isogenic derivative harboring a null deletion in *fliC* (Δ*fliC*) was constructed to serve as an amotile, aflagellated control. The genetic structure of these flagellated and aflagellated *S*. Typhi derivatives were confirmed by sequencing and the different derivatives were then screened in agglutination assays with flagella specific antisera and were found to express the appropriate flagella antigens (Figure 2).

All three *S*. Typhi derivatives harboring functional flagellin genes expressed peritrichous flagella observable by negative staining under the transmission electron microscope (TEM) (Figure 2a), whilst the Δ*fliC* had no detectable flagella. *S*. Typhi H*d* and H*j* expressing derivatives elaborated nine (range: H*d*; 3–18, H*j*; 5–16) and the H*z66* variant a median of seven flagella per cell (range; 4–14) (Figure 2b). The H*z66* flagella had a significantly greater diameter (median: 13.1 nm, range: 12.1–14.9 nm) than both the H*d* and H*j* flagella (median; 12.1 nm, range; 10.6–13.7 nm, and median; 8.74 nm, range: 8.19–9.77 nm respectively) (*p*< 0.0001; 2 sided *t*-test) (Figure 2c and 2d). Furthermore, the H*j* flagella were significantly shorter in length (median; 2.06 μm, range; 0.33–8.16 μm) than both the H*d* (median; 4.05 μm, range 0.45–10.2 μm) and H*z66* flagella (median; 4.65 μm, range: 0.545–10.3 μm) (*p*<0.0001; 2 sided *t*-test). The difference in length between the H*d* and the H*z66* flagella was not significant.

The motility of the *S*. Typhi flagella variants was measured by assessing their swimming capabilities in soft media over a defined incubation period. The flagellated *S*. Typhi Δ*fliC* derivative was amotile, while each of the flagellated *S*. Typhi swam between 37 and 58 mm in the agar matrix during the 16-hour incubation period at 37°C (Figure 2e). The H*z66*-expressing *S*. Typhi derivative was consistently the least motile. Despite having the shortest flagella, the H*j S.* Typhi derivative migrated significantly further in the soft agar (median; 57 mm, range; 57–58 mm) than the H*d* and the H*z66* derivatives (median; 50 mm, range; 49–51 mm, median; 38 mm, range 37–41 mm, respectively) (*p*<0.0001; 2 sided *t*-test).

### Indonesian Typhoid patients elaborate IgG against Hd, Hz66 and Hj flagellin

Flagellin is highly immunogenic and antibodies (IgG) against *S*. Typhi flagellin can be measured for a prolonged period after a confirmed typhoid infection^15^. As the isogenic *S*. Typhi derivatives demonstrated different phenotypic qualities we hypothesized that the organisms expressing these flagella antigens might stimulate different responses from the immune system during natural infection. To test this hypothesis, IgG against H*d*, H*j* and H*z66* flagellin was measured in a group of typhoid patients. Firstly, the type of flagella genes encoded in *S.* Typhi isolated during a typhoid case/control study conducted in Jakarta, Indonesia, a region where *S*. Typhi expressing the H*d*, H*j* and H*z66* flagella are co-circulating, were assessed by PCR amplification. Thirty *S*. Typhi isolates, where a corresponding acute serum sample from a typhoid patient was available, were analyzed and 15 were H*d*, 4 were H*d:*H*z66*, 11 were H*j*:H*z66*, and none were H*j* only (Table 1). Available disease metadata was stratified by flagellin variant and there was no significant difference between the three groups and the number of days of fever prior to hospitalization. However, H*z66*-positive *S*. Typhi originated from on average older patients than the isolates expressing H*d* alone by a mean of seven years (*p* = 0.014; 2 sided *t*-test).

**Figure 3:**
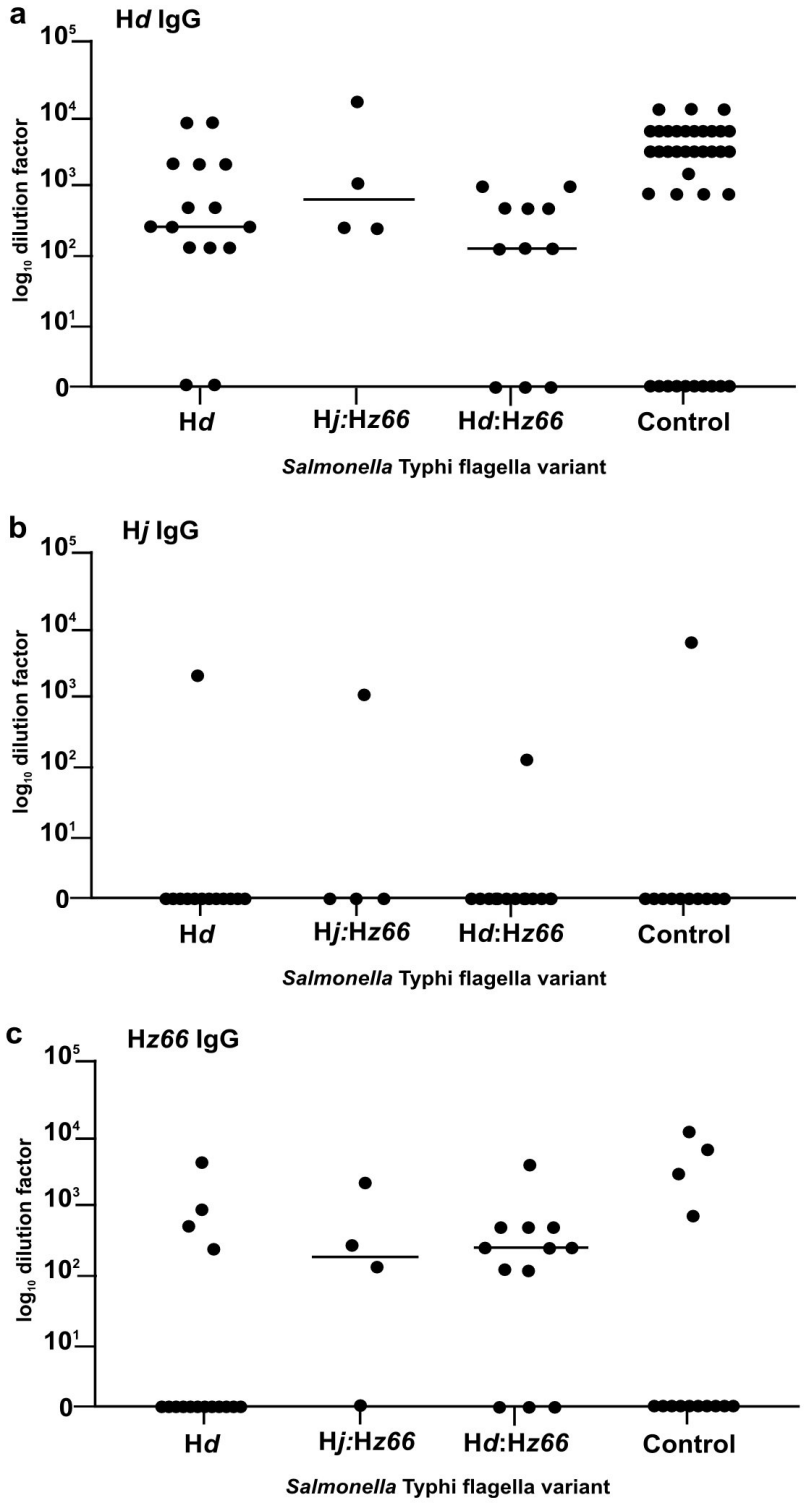
The antibody response to H*d*, H*j*, and H*z66* flagellin in Indonesian subjects. Anti-flagellin IgG antibody titers in serum from typhoid fever patients (n = 30; 15 H*d*, 4 H*j*:H*z66*, 11 H*d*:H*z66*) and community controls (n = 79) in Indonesia. a) Scatterplot of IgG measurements against H*d* flagellin in patients infected with H*d*, H*j*:H*z66*, or H*d*:H*z66* *S*. Typhi and controls. b) Scatterplot of IgG measurements against H*j* flagellin in patients infected with H*d*, H*j*:H*z66*, or H*d*:H*z66* *S*. Typhi and controls. c) Scatterplot of IgG measurements against H*z66* flagellin in patients infected with H*d*, H*j*:H*z66*, or H*d*:H*z66* *S*. Typhi and community controls. Titers measured as the log_10_ of the highest dilution with an OD three times the OD value of negative controls.

Serum from the 30 from typhoid patients from which the above *S*. Typhi flagella variants were isolated and 79 from asymptomatic controls (Table 1) were screened using an ELISA to measure IgG against the three forms of *S*. Typhi flagellin (Figure 3). The majority of typhoid patients (25/30), regardless of the *S*. Typhi flagella variant isolated from their blood, harbored IgG against H*d* (Figure 3a). Furthermore, the preponderance of patients (12/15) infected with an H*z66 S*. Typhi harbored IgG against H*z66,* as did four patients infected with H*d* isolates (Figure 3c). Conversely, only three patients demonstrated an anti-H*j* IgG response, of which only one was infected with an *S*. Typhi H*z66*:H*j* isolate (Figure 3b). Interestingly, the asymptomatic controls also harbored antibody responses of a similar magnitude to the typhoid fever patients, indicating probable previous exposure. Of the 79 asymptomatic controls, 31 (39.2%) harbored IgG against at least one of the flagellin antigens, with the majority responding to H*d* (30/31, 97%), four (5.1%) to H*z66* IgG with only one of these to H*z66* exclusively and also to H*d*. Only one control sample harbored measurable anti-H*j* IgG and this also harbored IgG against the other two flagellin antigens. We found no significant difference in IgG levels to H*d*, H*j,* or H*z66* flagellin between the asymptomatic controls or those infected with H*d*, H*j* or H*z66* *S*. Typhi (*p*>0.05 in all pairwise comparisons; 2 sided *t*-tests).

### S. Typhi flagella variants induce similar activation of TLR5

**Figure 4:**
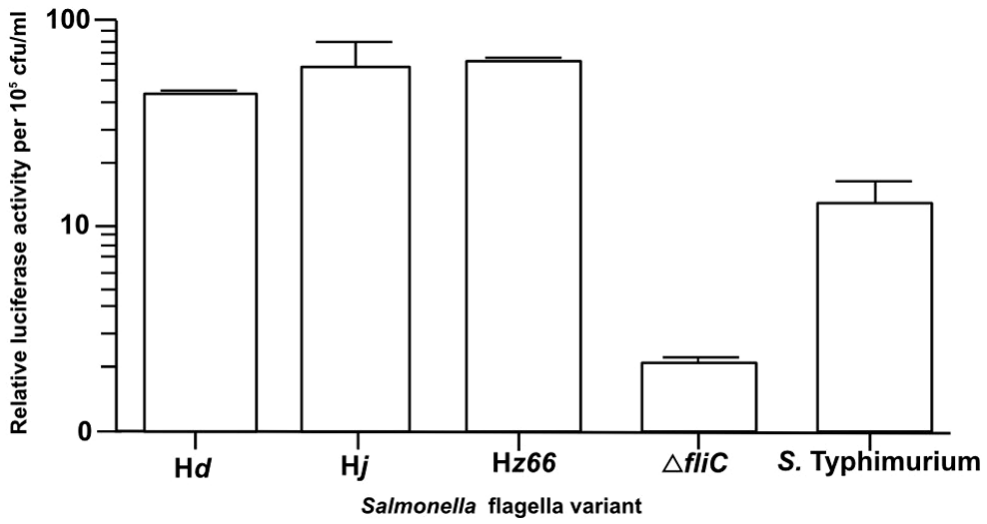
TLR5 activity induced by *S*. Typhi flagella derivatives. Histogram showing the relative ability of *S*. Typhi Hd, Hj, Hz66 and Δ*fliC* to induce TLR5 activity after bacterial inoculation on to transfected HEK293 cells compared to an *S*. Typhimurium positive control. Results are measured as NFκB-luciferase activity relative to Renilla-luciferase activity per 10^5^ cfu mL^−1^ of infecting bacteria.

Predicting that the *S*. Typhi flagella variants may have a differing ability to stimulate Toll Like Receptor 5 (TLR5), we independently co-transfected HEK293 cells expressing TLR5 with the three *S*. Typhi flagella variants, *S*. Typhi Δ*fliC* or *S.* Typhimurium SL1344 as a positive control (Figure 4). All of the *S*. Typhi derivatives, with notable exception of the Δ*fliC* mutant, were able to stimulate TLR5 and produce downstream NFκB signaling with a similar degree of potency.

### The interaction of S. Typhi flagella derivatives with macrophages and epithelial cells

**Figure 5:**
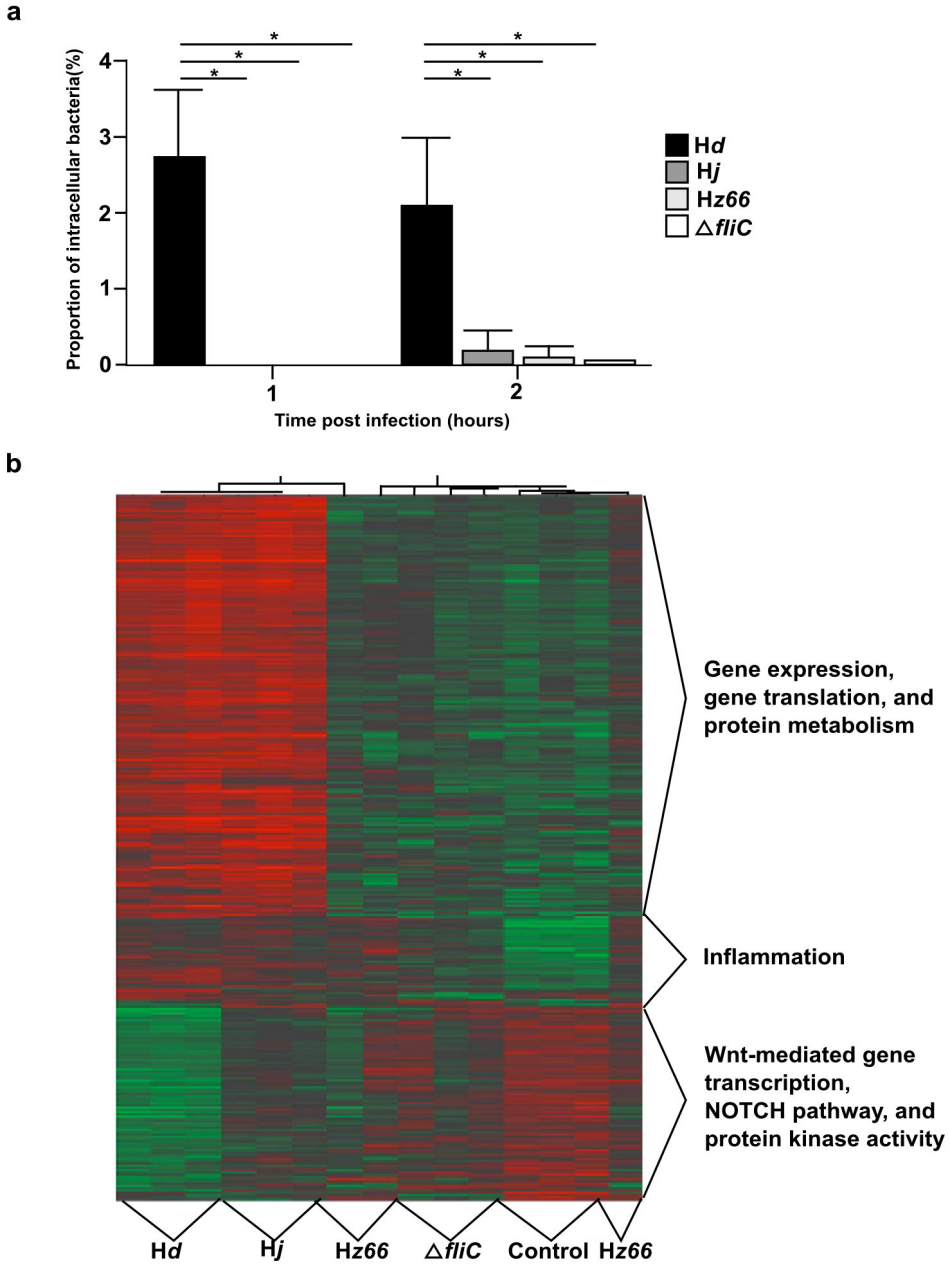
The interaction of *S*. Typhi flagella derivatives with macrophages. The moncytic THP-1 cell line was differentiated into macrophages and infected with *S.* Typhi expressing one of the three-flagellin variants and the non-flagellated mutant (Δ*fliC*) (a) Histogram showing the median proportion of recovered (intracellular) bacteria normalized by the inoculum and averaged over six experiments at 1 and 2 hours post infection. Asterisk highlights statistically significant variations by pairwise comparison (*p*<0.001), error bars represent one standard deviation. (b) THP-1 cells were infected and RNA was isolated for microarray analysis of host mRNA population. Figure shows hierarchical clustering of differentially expressed genes in THP-1 cells infected with the three flagellin derivatives, and the non-flagellated mutant (Δ*fliC*), compared with uninfected THP-1 cells. The cut-off for differentially expressed genes was an absolute fold change >2 and FDR corrected *p*-value of <0.05. Red/green color scale indicates level of gene expression, red indicates increased gene expression, and green indicates reduced gene expression.

The *S*. Typhi derivatives were inoculated onto THP-1 cells to measure cellular uptake and *S*. Typhi expressing the H*d* flagella were consistently taken up by more efficiently than the H*j* and H*z66* expressing derivatives (Figure 5a). The Δ*fliC* *S*. Typhi was reproducibly taken up at a lower frequency than any of the flagellated derivatives. No difference in cytotoxicity was observed between the *S*. Typhi derivatives using a lactate dehydrogenase assay (data not shown).

The transcriptome of THP-1 cells infected with the *S*. Typhi derivatives was measured by DNA microarray analysis of the host mRNA populations. *S*. Typhi expressing H*d* and H*j* flagellin induced highly similar transcription patterns in host cells, with higher numbers of differentially expressed genes, compared to the H*z66* or Δ*fliC* derivatives (Table 2). The pathway and gene ontologies of the differential expressed genes groups were determined using InnateDB and could be divided into three main groups with corresponding profiles (Figure 5b). These three main groups were; i) the up-regulation of genes involved in inflammation in cells infected with flagellated bacteria; ii) the up-regulation of gene involved in gene expression, translation, and protein metabolism in cells infected with either H*d* or H*j* derivatives and; iii) the proportional down-regulation of genes in the NOTCH pathway, Wnt-mediated gene transcription, and protein kinase activity with flagellated organisms.

**Figure 6:**
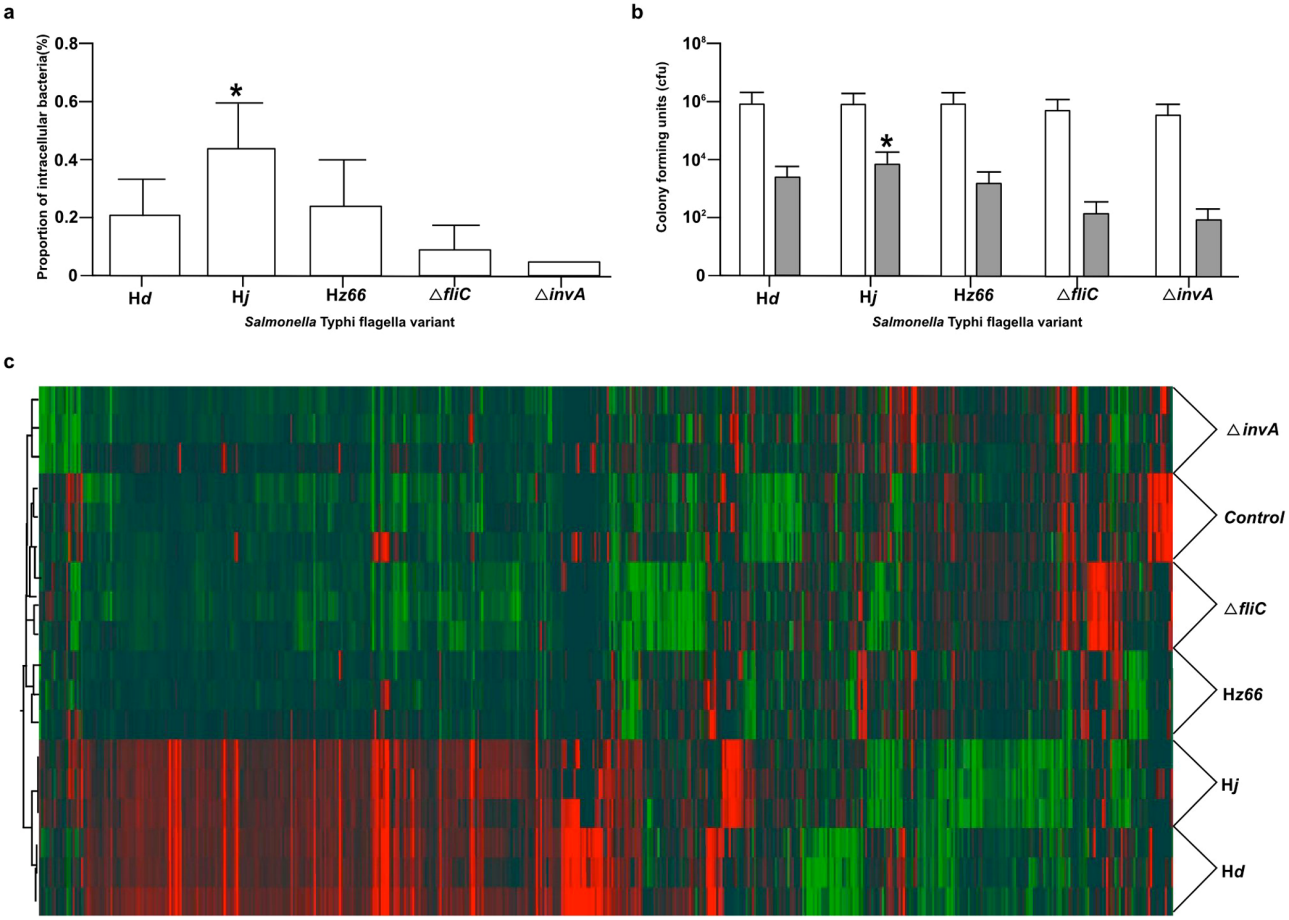
The interaction of *S*. Typhi flagella derivatives with epithelial cells. Hep-2 cells were infected with *S.* Typhi expressing one of three flagellin variants, a non-flagellated mutant (Δ*fliC*), and an invasion deficient mutant (Δi*nvA*). (a) Histogram showing the median proportion of recovered intracellular bacteria normalized by the inoculum and averaged over six experiments (*p = 0.0002, for H*j* compared to other variants by ANOVA). Error bars show 1 standard deviation from the mean. (b) Histogram showing the number of recovered bacteria during attachment (white columns) and invasion (grey columns) assays averaged over six experiments (*p = 0.0001, for H*j* compared to other variants by ANOVA). (c) Transcriptome analysis of infected Hep-2 cells. Figure shows hierarchical clustering of differentially expressed genes in Hep-2 cells infected with *S*. Typhi expressing one of the three flagellin variants, the non-flagellated mutant (Δ*fliC*), and the invasion deficient mutant (Δi*nvA*), compared with uninfected Hep-2 cells. The cut-off for differentially expressed genes was an absolute fold change >2 and FDR corrected *p*-value of <0.05. Red/green color scale indicates level of gene expression, red indicates increased gene expression, and green indicates reduced gene expression.

Next, the various flagella variants were independently inoculated onto human epithelial-like Hep-2 cells (Figure 6). The H*d* and H*z66* *S*. Typhi derivatives invade Hep-2 cells with a comparable frequency but the *S*. Typhi H*j* derivative reproducibly demonstrated a significantly higher capability for invasion (0.42% ± 0.15) (Figure 6a). Furthermore, *S*. Typhi Δ*fliC* was reproducibly even less invasive and was only marginally more invasive than *S*. Typhi Δ*invA*. We found no significant difference in the ability of the various flagellated derivatives or Δ*fliC* and Δ*invA* *S*. Typhi to attach to the epithelial cells (Figure 6b).

Similar to the work on THP-1 cells, gene expression profiles were determined using microarray analysis of mRNA populations present in epithelial cells exposed to *S*. Typhi expressing the different flagellins. Levels of IL8 mRNA and pathways related to cytokine-cytokine receptor interactions and the HIF1α transcription factor network were comparatively over expressed by Hep-2 cells exposed to flagellated *S*. Typhi compared to the Δ*fliC* derivative. Unsupervised hierarchical clustering revealed a similar response to *S*. Typhi expressing either H*d* or H*j* but this was distinct from *S*. Typhi H*z66* (Figure 6c). In fact, the overall host transcriptome response to the H*z66* was more comparable to the response induced by *S*. Typhi Δ*fliC* and Δ*invA* than the H*d* and H*j* derivatives (Table 2). This difference was mainly restricted to the up-regulation of genes involved in gene expression, translation and general protein metabolism e.g. genes CDK1, CDKN1B, MNAT1, CCNG1, CCNG2, RPL7, RPL9, RPL14 and MYC (Supplementary information).

**Figure 7:**
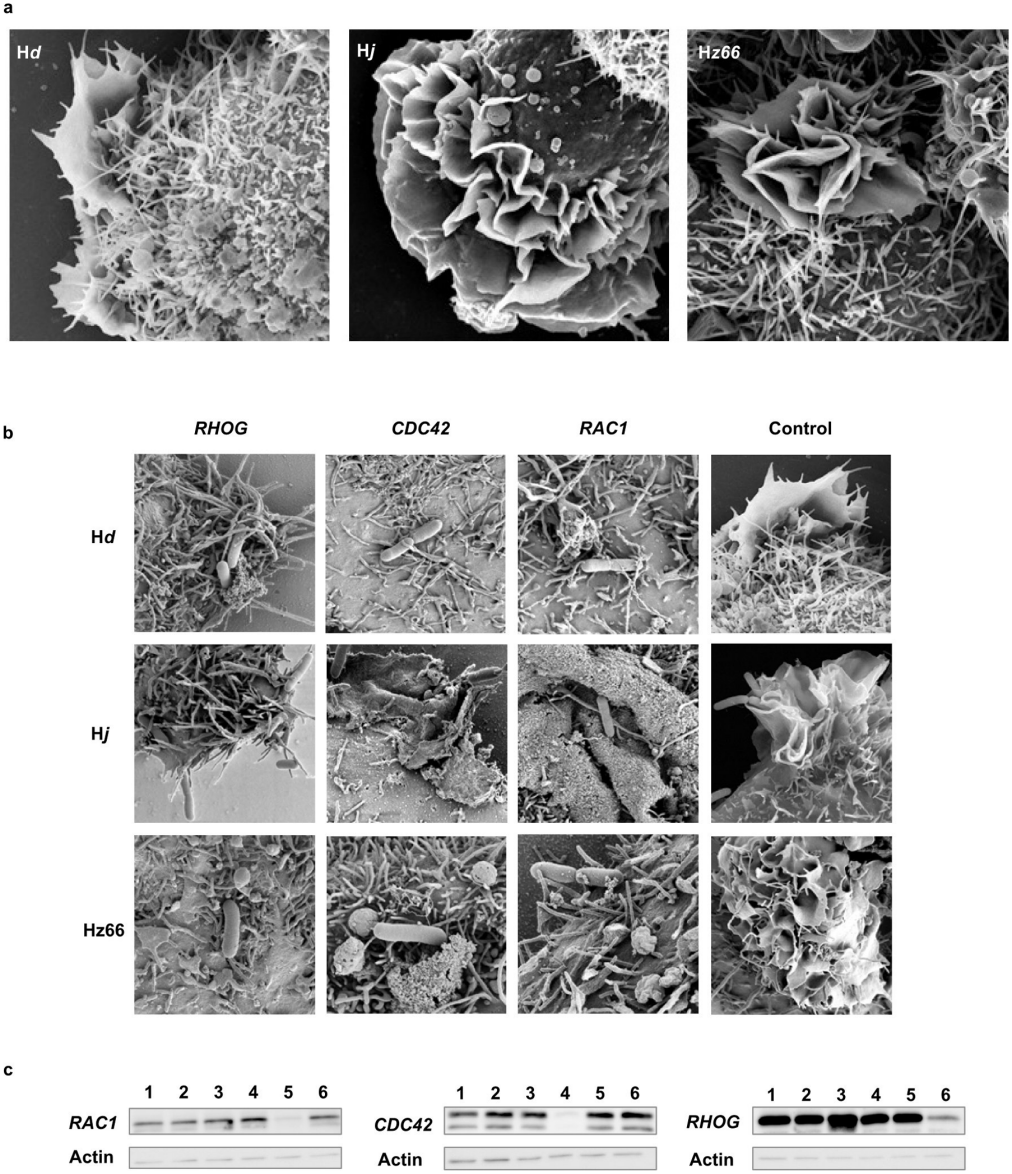
The ability of *S*. Typhi flagella derivatives to induce ruffling in epithelial cells. (a) SEM images of Hep-2 cells infected with *S*. Typhi H*d*, H*j,* and H*z66* flagella derivatives. Images show ruffles induced by *S*. Typhi inducing cytoskeletal rearrangement, exaggerated by *S*. Typhi H*j*. (b) The role of Rho GTPases during ruffle formation was assessed by using iRNA to block the RAC1, CDC42, and RHOG Rho GTPases. Images prepared by SEM of Hep-2 cells with knock down Rho GTPases or controls infected with *S.* Typhi*.* (c) Western blot of cell lysates, confirming the lack of Rho GTPases expression after iRNA. Lanes, 1; untreated cells, 2; reagent control, 3; non-interfering siRNA control, 4; CDC42 siRNA, 5; RAC1 siRNA and 6; RHOG siRNA.

During epithelial cell invasion, *Salmonella* trigger rearrangements of the host cell cytoskeleton including ruffles in the plasma membrane. SEM was used to compare the interaction of *S*. Typhi expressing H*d*, H*j* or H*z66* flagellin with epithelial cells during invasion and to gain insight into the different gene expression profiles. All *S*. Typhi were able to stimulate substantial membrane ruffling but abnormally large ruffles were consistently observed when *S*. Typhi expressing H*j* flagella interacted with epithelial cells (Figure 7a).

Ruffles are triggered in part by effectors secreted through the Salmonella Pathogenicity Island I (SPI1) interacting with host Rho-GTPases, including Rac1, Cdc42, and RhoG^16^,^17^,^18^. To assess the contribution of each of these Rho-GTPases to ruffling, siRNAs were generated for each gene and ruffling was observed on Hep-2 cells exposed to individual siRNAs using TEM. siRNAs to RAC1, CDC42 or RHOG completely repressed ruffle formation on Hep-2 cells exposed to *S*. Typhi expressing the H*d* or H*z66* flagella (Figure 7b). Cell ruffling was reduced when H*j S*. Typhi were inoculated onto Hep-2 exposed to RAC1 but interestingly not RHOG and CDC42 siRNAs.

## Discussion

We performed a series of experiments using a combination of typhoid patients and in vitro assays to assess the impact of flagella type on host cell-pathogen interactions involving *S*. Typhi. To facilitate these studies we constructed a novel series of carefully engineered isogenic *S*. Typhi derivatives differing only in the antigenic structure of their flagella. These data are of interest as the majority of global *S*. Typhi isolates are monophasic and express only the classical H*d* flagella yet novel *S*. Typhi are originating in the Indonesian archipelago that can express alternative flagellin either from an allelic variant of H*d* known as H*j* or from a novel *fljB* gene encoded on a linear plasmid pBSSB1, known as H*z66*^13^,^14^. Here we show that the different flagellin have distinct structural features that directly impinge on the motility of the bacteria and their pathogenic potential. Firstly, we found that H*z66* flagella were measurably thicker than H*j* or H*d* flagella and that this property translated into poorer motility when comparing *S*. Typhi Hz66 to H*d* and H*j*-positive derivatives. Correspondingly, *S*. Typhi H*j* produced shorter and thinner flagella structures and swam faster than both H*d* and H*z66 S*. Typhi derivatives. During laboratory observation the H*j* flagella were found to be more fragile that the other flagella variants and were detected mainly in the culture medium whereas the other flagella were predominantly attached to the bacterial body. This data are broadly in keeping with previous observations on *S.* Typhimurium flagellin genes with deletions approximately the same size and location as H*j*^19^. However, our results are somewhat different some previously published observations, in which non-isogenic H*d* strains were reported to have a higher motility^20^ but here isogenic derivatives were used to compare these phenotypic characteristics.

Data collected using Hep-2 or THP-1 cells indicated that the type of expressed *S*. Typhi flagella can dramatically influence host/pathogen interactions. These results were supported by our unpublished observation that S. Typhi Δ*flgK* derivatives that produce and secrete unpolymerized flagellin also exhibit a reduced ability to invade Hep-2 cells, in keeping with the impact of flagellin in *S.* Typhimurium pathogenesis^21^,^22^. The increased capacity of the *S*. Typhi H*j* to invade Hep-2 cells appears to correlate with the induction of larger cellular actin ruffles than those induced by either H*d* or H*z66 S*. Typhi derivatives. The formation of these ruffles is associated with invasion, triggered when *Salmonella* have intimate contact with a non-phagocytic cell and involves a number of *Salmonella* Pathogenicity Island associated effector proteins^23^. These effectors interact with Rho GTPases within the host cell, stimulating a rearrangement of actin in the host cell cytoskeleton^16^,^17^,^18^. To investigate the mechanism of ruffle formation we performed a number of iRNA experiments to suppress the expression of Rho GTPases, which are known to interact with *Salmonella* effectors (RHOG, RAC1, CDC42). The resulting data demonstrated that the dramatic ruffle formation induced by *S*. Typhi H*j* occurred independently of CDC42 and RAC1. These data predict the existence of an alternative signaling cascade resulting in bacterial internalization, which is activated through contact with bacterial flagellin. In support of this hypothesis, it has been shown that *S.* Typhimurium flagellin is injected into the host cell cytosol in part through SPI1^24^, and that flagellin may be acting as an ‘effector protein' during cellular invasion and uptake.

As with our observations in epithelial cells, the presence of flagella was required for the efficient uptake of *S*. Typhi into macrophages. Here, we also observed differences in invasion rates between the *S*. Typhi flagellated derivatives but here the *S*. Typhi H*d* derivative had a greater capacity for internalization than the *S*. Typhi H*j*. This differential interaction between the H*d* and H*j* derivatives with macrophages may, in part, be one of the factors influencing why H*d S*. Typhi are successfully globally whereas H*j* derivatives predominantly restricted to the islands of Indonesia. However, additional factors such as host genetics and environmental conditions may play an even greater role in this atypical geographic restriction. Others have suggested a role for the predatory protozoan species carried by Indonesians^25^. A role for immune invasion in the evolution of the novel Indonesian *S*. Typhi remains unproven, although the apparent poor immunogenicity of H*j* may be contributing to local selection on *S*. Typhi. Here, we speculate that the H*z66* variant is moving into a niche in individuals who have been exposed to H*d* *S*. Typhi.

We found a wide variation in gene expression patterns between cells exposed to the *S*. Typhi flagella derivatives. For example, the *S.* Typhi H*z66* derivative consistently induced transcriptome profile more similar to Δ*fliC* and Δ*invA* than the H*d* and H*j* *S*. Typhi, which were in turn more similar. All the flagellated bacteria, however, induced a robust and measurable acute inflammatory response, in keeping with the lack of detected difference in signaling through TLR5.

Indonesia is still endemic for typhoid fever, with an estimated incidence of 810/100,000 cases per year^26^. The number of individuals within the community with a substantial IgG response to *S*. Typhi flagellin presumably reflects this high prevalence of typhoid in Indonesia. The serum from several individuals (both typhoid fever patients and community controls) exhibited signatures correlating with multiple infections with *S*. Typhi expressing different flagella antigens. This theory of multiple infections is supported by the difference in median ages, since those infected with H*j* or H*z66* *S*. Typhi were significantly (for H*z66*) older than those with H*d* infections. These data suggest that H*z66* *S*. Typhi may be more ‘opportunistic' than *S*. Typhi H*d*, exploiting a niche after a previous infection/exposure to *S*. Typhi H*d*. An additional observation was that both H*d* and H*z66* flagellin appears to induce a more robust IgG response than H*j*. It is noteworthy that when mice were immunized with H*j* flagellin they mounted a much weaker antibody response compared to similar mice immunized with H*z66* or H*d* flagellin (our unpublished observations). This reduced immunogenicity of H*j* flagella is perhaps not surprising, since the single dominant B cell epitope of H*d* is centered at residues 229–230, within in the section missing in H*j* (aa 224–310)^27^.

In summary, the data presented here indicates the important, active role of flagella in host pathogen interactions during *S.* Typhi infection, engaging both innate and adaptive branches of the immune response. The differences in invasion and immunogenicity observed between flagellin variants suggests an almost opportunistic behavior of the less widespread variants (H*j* and H*z66*), taking advantage of preexisting anti-H*d* immunity; an issue that should be taken into account when developing novel whole-cell flagellated *Salmonella* vaccines.

## Experimental procedures

### Bacterial and genetic manipulation

The attenuated *S.* Typhi Ty2 derivative BRD948 (H*d*), harboring deletions in the *aroA, aroC* and *htrA* genes, was used for all experiments to avoid safety issues related to genetically engineering a containment level three organism^28^. *S.* Typhi BRD948 and derivatives are approved for use in a containment level two laboratory in the United Kingdom^29^. All genetic manipulations were performed using Luria–Bertani (LB) media supplemented with 40 mg L^−1^ of l-phenylalanine and l-tryptophan, and 10 mg L^−1^ of p-aminobenzoic acid and 2,3-dihydroxybenzoic acid (*aro* mix). When required, media was supplemented with chloramphenicol, ampicillin or kanamycin and growth temperatures were adjusted (37°C or 42°C), according to the requirements for the elimination of plasmids through temperature sensitive replication. Isogenic *S.* Typhi flagella variants (H*d*, H*j* and H*z66*) were constructed using the lambda red recombinase method^30^. Firstly, a *fliC* (non-motile) mutant was constructed. PCR amplicons were designed and produced to remove *fliC* in its entirety but leaving the upstream and downstream regions intact. Plasmid pKD3 was used as a template for PCR amplifications. PCR amplicons were electrotransformed in *S.* Typhi BRD948 pKD46 as previously described^29^. A non-flagellated mutant derivative, *S.* Typhi BRD948 Δ*fliC*, was generated after selection with chloramphenicol and screened for a lack of motility using 0.3% LB swim agar plates with appropriate supplementation. The pKD3 insertion, including the antimicrobial resistance cassette, was removed using pCP20, as previously described [2]. H*j* and H*z66* flagellin variants were constructed by PCR amplification of the H*j* and H*z66* loci (and 200 bp of upstream and downstream flanking sequence) from strain *S*. Typhi Ty404^31^. PCR amplicons were electrotransformed in *S.* Typhi BRD948 Δ*fliC* containing pKD46 as before. Organisms ‘complemented *in situ*' with alternative flagellin genes were selected on the basis of their ability to swim in 0.3% LB swim agar plates with appropriate supplementation. Motile organisms were subcultured to ensure purity and screened by PCR amplification and sequencing to ensure the H*j* and H*z66* encoding genes were inserted correctly.

### Cellular invasion assays

Hep-2 cells were grown in Dulbecco's Modified Eagle Medium (DMEM, Sigma), 10% fetal calf serum (FCS, Sigma) and 2 mM L-glutamine (Sigma), at 37°C in 5% CO_2_. The day before the infection assays were performed, cells were seeded at 1 × 10^5^ cells/well in 24-well plates and incubated overnight. Bacteria were added at a multiplicity of infection (MOI) of 10:1 (bacteria:cell). Plates were then centrifuged at 600xG for 5 minutes to ensure contact with cells. Cells were incubated for 2 hours, washed with PBS and fresh medium supplemented with 100 μg mL^−1^ gentamycin was added. Cells were further incubated for an additional three hours. After washing, cells were lysed with 100 μl/well of 1% Triton X-100. Serial dilutions were performed and plated on agar plates for enumeration after overnight incubation.

For adhesion/invasion assays, Hep-2 cells were incubated at 4°C for 20 minutes prior to infection. Bacteria were resuspended in ice-cold DMEM and added at a MOI of 100:1. Plates were incubated for 1 hour at 4°C to allow attachment but not invasion; cells were then washed and lysed as described above. For the invasion assays, plates were washed and fresh pre-warmed DMEM was added to the wells. Cells were then incubated for one hour at 37°C. The media was then changed to DMEM with gentamycin and cells were incubated for another hour, before washing and cell lysis as described above.

THP-1 cells were cultured in RPMI-1640 medium (Sigma), containing 10% FCS, 2 mM L-glutamine, at 37°C and in 5% CO_2_. A week prior to infection, THP-1 cells were seeded onto 24-well plates, 1 × 10^5^ cells/well, and differentiated into macrophages with 50 ng mL^−1^ of phorbol 12-myristate 13-acetate (PMA, Sigma). Before adding the bacteria, cells were washed with PBS and fresh RPMI was added. Infections performed as described above. Cells were incubated for 30 minutes; then media was changed to RPMI supplemented with gentamycin. The cells were then incubated further for selected periods of time and then washed and lysed as described for Hep-2 cells.

### Electron microscopy

For microscopic analysis of infected cells, cells were seeded onto glass coverslips and infections were performed as described above. Samples were prepared for scanning electron microscopy (SEM) as previously described^32^ and for transmission electron microscopy (TEM) negative staining as previously described^33^. Images were collected using a 120 kV FEI Spirit Biotwin with a Tietz F4.15 CCD camera and the flagellum dimensions calculated using version 3 of TEM Tecnai software.

### Ruffling signaling cascade

A day prior to transfection, 2 × 10^4^ Hep-2 cells per well were seeded into 24-well plates. Immediately prior to transfection, fresh media was added to the wells. Transfections were performed using Lipofectamine™ RNAiMAX (Invitrogen), as per manufacturer instructions. Cells were transfected with 3 pmol siRNA (Dharmacon, RAC1: pool of D-003560-05, D-003560-07, D-003560-08, D-003560-09; CDC42: pool of D-005057-01, D-005057-02, D-005057-03, D-005057-04; RHOG: pool of D-008995-01, D-008995-02, D-008995-03, D-008995-0; siRNA control: ON-TARGET*plus* Non-Targeting Pool, D-001810-10) for 48 hours. After successful transfection, cells were infected with *S*. Typhi as described above for a period of one hour. After infection, cells were washed with PBS and processed for Western blotting and SEM. For Western blotting, cells were lysed with 100 μL cells lysis buffer (10 mM Tris-Cl pH 7.6, 5 mM EDTA, 150 mM NaCl, 0.5% Triton X-100, complete mini EDTA-free protease inhibitor (Roche)) and then centrifuged for 10 minutes at 14,000 rpm at 4°C. The supernatant was recovered and mixed with loading buffer. SDS-PAGE was performed using 15% acrylamide gels. Membranes were blocked in 0.05% PBS and 5% Tween BSA. Anti-RAC1 (1/1,000, mouse, Millipore); anti-CDC42 (1/1,000, rabbit, Cell Signalling Technologies); anti-RHOG (1/250, rabbit, Santa Cruz Biotechnologies) and anti-actin (1/10,000, rabbit, Sigma) were used as primary antibodies.

### Microarray analysis

Both THP-1 and Hep-2 cells were seeded and infected with *S*. Typhi as described above for a period of one hour. The strains used for infection were BRD948, BRD948-H*j*, BRD948-H*z66*, BRD948-Δ*fliC* and, in the case of Hep-2 cells, BRD948-Δ*invA*. An uninfected control well was also included. After infection, the cells were washed and RNA was purified using the RNeasy Mini Kit (Qiagen), as per manufacturer's instructions. RNA samples were then amplified and labeled using Illumina TotalPrep 96 kit (Ambion, Austin, TX, USA) and hybridized onto Illumina™ Human HT-12_V4 Beadchips (Illumina, San Diego, CA, USA). The chips were scanned on an Illumina BeadArray Reader and raw intensities were extracted using Illumina BeadStudio Gene Expression Module. Microarray data are available in the ArrayExpress database (www.ebi.ac.uk/arrayexpress) under accession number E-MTAB-2395.

Normalization and data analysis of the microarrays were performed using GeneSpring X software (Agilent Technologies). A quantile normalization using a baseline correction from the median of all samples was performed. For each comparison, differentially expressed genes were defined as those exhibiting a fold change ≥ 2 and a FDR (false discovery rate) corrected *p-*value ≤ 0.05. Adjusted *p-*values were calculated using the Benjamini and Hochberg method^34^. Pathway, gene ontology (GO), and interaction analysis was performed using InnateDB (www.innatedb.ca). Over-represented pathways or GO terms were deemed significant if having a FDR corrected *p-*value ≤ 0.05.

### TLR5 signaling

TLR5 signaling was measured by using HEK293 cells (human embryonic kidney cells, ATCC number CRL-1573) transfected with a TLR5 expression vector, an NFκB-luciferase reporter (firefly luciferase, Stratagene) a Renilla-luciferase reporter as a transfection control (Promega), and a ‘filler' plasmid pEF-BOS, using Fugene 6 (Roche). Twenty-four hours after transfection, the cells were incubated with serially diluted overnight cultures of the relevant bacteria (BRD948-H*d*, BRD948-H*j*, BRD948-H*z66,*BRD948- Δ*fliC* and *Salmonella* Typhimurium UK1 (positive control)). After six hours infection, the cells were washed and incubated with firefly and TK-Renilla luciferase substrates. Cells were then infected with Luciferase activity was measured using the Luciferase Assay System (Promega) as per manufacturer's instructions. The optimal bacterial dilution was defined as the dilution at which the greatest differential in the expression of the two luciferases for the positive control was provided. The same bacterial concentrations for the different mutants were then compared for pNFkB-luciferase expression; results are presented as NFκB-luciferase activity relative to Renilla-luciferase activity.

### Anti-flagellin ELISA

The metadata associated with the available serum samples for serology are as previously described^26^,^31^,^35^. Briefly, the serum from the typhoid cases with a range of *S*. Typhi variants was collected as part of a case/control study conducted by Vollaard et al.^26^, all these patients had positive blood-culture containing *S.* Typhi. Community controls were selected from the local community at random and as previously described^35^. Strains isolated from blood cultures of typhoid patients were assessed by PCR amplification to detect the nature of the native flagellin gene(s)^31^. For this work, thirty typhoid cases with known flagella variants and 79 randomly chosen community controls were selected for serological analysis.

For the ELISA assays, Nunc MaxiSorp (Thermo Scientific) or Microlon (Greiner) 96-well plates were coated with 2 μg mL^−1^ of *S.* Typhi flagella antigens (H*d*, H*j* or H*z66*, purified as described previously) in phosphate buffer pH 9.5 and incubated overnight at 4°C. The plates were blocked with 1% BSA (Sigma) in PBS-0.05% Tween (Sigma) for 1 hour at 37°C. Sera were added in serial dilutions in PBS-0.05% Tween-0.1% BSA and incubated for two hours at 37°C. The secondary antibodies (rabbit anti-human IgG-HRP conjugated (Dako), mouse anti-human IgG1-biotin conjugated (Sigma) or mouse anti-human IgG2-biotin conjugated (Sigma)) were added at a 1/1,000 dilution in PBS-0.05% Tween-0.1% BSA and incubated for two hours at 37°C. Plates were developed with OPD (SIGMA*FAST*™ OPD, Sigma) at room temperature for 10 minutes, following the manufacturers' instructions. The reaction was stopped with 20 μl/well 3 M H_2_SO_4_. Plates were read at 490 nm using an ELISA plate reader.

### Statistical analysis

Statistical tests were applied to determine differences between the strains with respect to flagella morphology, motility, and cellular invasion. The nature of these tests is outlined in the results with the corresponding *p-*value. ANOVA and 2-sided t-tests were performed using GraphPad Prism (GraphPad Software Inc.), for multiple of pairwise comparisons, respectively. No correction for multiple testing was applied. *p* values of ≤ 0.05 were considered to be statistically significant.

## Author Contributions

This project was conceived by F.S., R.A.K., D.G., G.D., S.B., the experiments were performed by F.S., S.K., R.A.K., S.C., S.B., material, expertise of reagents contributing to this work were supplied by G.F., S.C., D.G., VvdV, J.T.v.D., R.S., G.T., the manuscript was drafted by F.S., R.A.K., G.D., S.B. and all authors (F.S., S.K., G.F., S.C., D.G., V.v.d.V., J.T.v.D., R.S., G.T., R.A.K., G.D., S.B.) provided critical input and approved the final manuscript of the manuscript.

## Figures and Tables

**Table 1:** The characterics of control subjects and typhoid fever cases infected with *S*. Typhi expressing combinations of the three flagella variants

Group	N	Male sex (%)	Median age in years (range)	Median days of fever (range)
Typhoid with *S*. Typhi H*d*	15	8 (53)	19 (10–35)	5 (3–14)
Typhoid with *S*. Typhi H*d*:H*z66*	4	2 (50)	23.5 (15–30)	5 (5)
Typhoid with *S*. Typhi H*j*:H*z66*	11	7 (64)	29 (11–57)	4 (3–30)
Community controls	79	23 (29)	24 (7–74)	NA

**Table 2:** The differential gene expression changes induced by *S.* Typhi flagella derivatives in THP1 and Hep2 cells

*S*. Typhi type		THP1			Hep2	
	Up regulated*	Down regulated	Total	Up regulated	Down regulated	Total
**H*d***	200	100	300	537	169	706
**H*j***	228	5	233	480	220	700
**H*z66***	32	9	41	153	120	273
**Δ*fliC***	16	8	24	117	196	313
**Δi*nvA***	NA	NA	NA	129	132	261

*Table shows the number of differentially expressed genes in infected cells, compared to an uninfected control. The cut off values for differentially expressed genes is a ≥2 fold change and an FDR corrected *p-*value of ≤ 0.05.
